# Differential Diagnosis of Kleine-Levin Syndrome

**DOI:** 10.15844/pedneurbriefs-29-3-7

**Published:** 2015-03

**Authors:** J. Gordon Millichap

**Affiliations:** 1 Division of Neurology, Ann & Robert H. Lurie Children's Hospital of Chicago, Chicago, IL; 2 Departments of Pediatrics and Neurology, Northwestern University Feinberg School of Medicine, Chicago, IL

**Keywords:** Kleine-Levin syndrome, Hypersomnia, Differential diagnosis

## Abstract

Investigators at Pitie-Salpetriere and Robert Debre Hospitals, and other centers in France, evaluated consecutive patients referred for suspected Kleine-Levin (KLS) syndrome, detailed differential diagnoses, and examined characteristics of patients with prolonged (>30 days) episodes.

Investigators at Pitie-Salpetriere and Robert Debre Hospitals, and other centers in France, evaluated consecutive patients referred for suspected Kleine-Levin (KLS) syndrome, detailed differential diagnoses, and examined characteristics of patients with prolonged (>30 days) episodes. Of 166 referred patients, 120 had typical primary KLS syndrome (secondary to brain diseases, n=4, atypical syndrome, n=7, mostly psychiatric, n=29, incomplete data, n=6). Patients were more male (64%) than female (p=0.002), most were Caucasian whereas Jewish origin was exceptional (5%), they were more often of premature birth, had more frequent birth and developmental abnormalities (45%) than controls, most (80%) were teenagers at onset (10% were <12 years), and a family history of epilepsy was more frequent than in controls. Initial episodes were prolonged (32 vs 11 days) in 34 and short in 85. Patients with prolonged episodes were older at first interview than those with short episodes, and the disease course was longer (9 vs 6 years). During episodes, patients with prolonged episodes had shorter sleep time, higher levels of anxiety and agitation, and more feelings of disembodiment and amnesia. Between episodes, they were more tired, needed more naps, fell asleep more rapidly, and had higher anxiety/depression scores. The most frequent symptoms were sleep disorders, cognitive impairment, altered perception, eating disorders (more or less than normal), and apathy. Mental disorders are frequent differential diagnoses. An infection (URI, gastroenteritis, mononucleosis, H1N1 influenza) was present in 42% of patients at KLS onset. Other triggering factors included vaccination, sleep deprivation, stress, mental effort, alcohol, traveling, menses, marijuana, and head trauma. [1]

COMMENTARY. The eponym “Kleine-Levin syndrome” as defined above [1] was coined by Critchley and Hoffman [2] in 1942. The diagnostic characteristics are episodic hypersomnia, altered mental state, cognitive impairment, hallucinations, delusions, hyperphagia, and hypersexual behavior during episodes. Data from a multinational series of 108 patients [3] highlight hypersomnia, cognitive disturbance, and derealization as core symptoms, compared to less frequent symptoms of disinhibited behavior, including megaphagia in 60% and hypersexuality in 30%. Suspected causes include hypothalamic dysfunction or autoimmune disorder, and precipitants are infection, alcohol or head trauma. No known genetic marker is described. The diagnosis is by exclusion, ruling out narcolepsy, temporal lobe epilepsy, Kluver-Bucy syndrome, metabolic disorders, bipolar and other mental disorders, and MS. The emphasis of cases with a prolonged course and residual abnormalities including apathy contradicts the concept of a generally benign disorder and warrants further diagnostic testing and early treatment trials [1]. A PubMed search uncovers several reports of KLS following viral encephalitis [4] and occasional cases that responded to antiepileptic medications (carbamazepine, valproate, and lamotrigine).
